# New Polymeric Adsorbents Functionalized with Aminobenzoic Groups for the Removal of Residual Antibiotics

**DOI:** 10.3390/molecules27092894

**Published:** 2022-04-30

**Authors:** Radu Ardelean, Adriana Popa, Ecaterina Stela Drăgan, Corneliu-Mircea Davidescu, Maria Ignat

**Affiliations:** 1 Industrial Chemistry and Environmental Engineering Faculty, Politehnica University Timișoara, 6 Vasile Parvan Blv., 300223 Timisoara, Romania; radu.ardelean@upt.ro; 2 “Coriolan Drăgulescu” Institute of Chemistry, 24 Mihai Viteazul Blv., 300223 Timisoara, Romania; apopa_ro@yahoo.com or; 3 “Petru Poni” Institute of Macromolecular Chemistry, 41A Aleea Grigore Ghica Vodă, 700487 Iași, Romania; 4 Research Institute for Renewable Energies (ICER), Politehnica University Timișoara, 138 Gavril Musicescu Street, 300501 Timișoara, Romania; 5 Faculty of Chemistry, “Al. I. Cuza” University of Iași, Carol I Bd. 11, 700506 Iași, Romania; mary_rud@yahoo.com

**Keywords:** adsorption, aminobenzoic acid, cross-linked copolymers, tetracycline, sulfamethoxazole

## Abstract

In this paper, we present the synthesis of new polymeric adsorbents derived from macroporous chloromethylated styrene–divinylbenzene (DVB) copolymers with different cross-linking degrees functionalized with the following aminobenzoic groups: styrene—6.7% DVB (PAB1), styrene—10% DVB (PAB2), and styrene—15% DVB (PAB3). The new polymeric products, PAB1, PAB2, and PAB3, were characterized by FTIR spectroscopy, thermogravimetric analysis, and EDX, SEM, and BET analysis, respectively. The evolution of the functionalization reaction was followed by FTIR spectroscopy, which revealed a decrease in the intensity of the γCH_2_Cl band at 1260 cm^−1^, and, simultaneously, the appearance of C=O carboxylic bands from 1685–1695 cm^−1^ and at 1748 cm^−1^. The thermal stability increased with the increase in the cross-linking degree. The data obtained from the EDX analysis of the novel cross-linked copolymers confirmed the functionalization with aminobenzoic groups through the presence and content of nitrogen, as follows: PAB1: N% = 0.47; PAB2: N% = 0.85; and PAB3: N% = 1.30. The adsorption performances of the novel polymeric adsorbents, PAB1, PAB2, and PAB3, were tested in the adsorption of three antibiotics, tetracycline, sulfamethoxazole, and amoxicillin, from aqueous solutions, by using extensive kinetic, equilibrium, and thermodynamic studies. The best adsorption capacity was demonstrated by the tetracycline. Amoxicillin adsorption was also attempted, but it did not show positive results.

## 1. Introduction

In recent decades, the global use of pharmaceutical active compounds, especially antibiotics, in order to improve human and animal health, has greatly increased, and their presence in the environment has led to the contamination of both surface water and groundwater [1]. Fleming’s discovery of penicillin in 1929 opened the era of antibiotics [2]. Antibiotics, as pharmaceutical agents, are widely used in medicine in the treatment of infectious diseases in both humans and animals, in aquaculture, and in other industries. Their frequent and widespread use causes both inefficiency in treatment and environmental problems due to their residual accumulation in the environment and resulting in microbial resistance [1,3]. The accumulation of antibiotics in wastewater treatment plants could pose a threat to human health and to the balance of the ecosystem due to their persistence, potential toxicity, and residues, which are considered persistent organic pollutants [3,4]. Various antibiotics have already been detected in groundwater, surface water, and hospital effluents, such as amoxicillin, sulfamethoxazole, and tetracycline, with concentration values in the range of ng/L to μg/L [5,6]. Sulfamethoxazole (SMX) is a representative antibiotic of the sulfonamide family, which is a group of veterinary pharmaceuticals. It is mainly given to patients who suffer from infectious diseases caused by bacteria [7]. Tetracycline (TC) is a widely used antibiotic because it has a broad spectrum of action against bacteria (both Gram-positive and Gram-negative), mycoplasmas, fungi (chlamydia), rickettsiae, and vermin, low toxicity and cost, and good oral absorption [8,9,10].

Several treatment methods have been developed to remove antibiotics from aqueous solutions, such as adsorption methods, biological treatment, coagulation–precipitation, electrochemical processes, ion exchange, membrane processes, and photocatalysis [9,10,11]. Among these methods, adsorption technology is still the most studied option for reducing antibiotics [12,13,14,15], since it is cost-effective and simple and highly reproducible, and a wide range of adsorbents is available [16,17,18].

Over time, researchers have effectively used different types of adsorbent material, such as carbon-based materials [19], polymers and resins [20,21,22], metal–organic frameworks [23], clays and minerals [24], zeolites [25], etc., to reduce the concentration of residual antibiotics in polluted waters.

The functionalization of polymeric supports with active pendant groups has provided structures with improved properties that can be used in a wide range of applications. The applications of these materials include their use as catalysts [26,27], antibacterial agents [28,29], and adsorbents for a wide range of pollutants [30,31]. Various polymers and resins have been successfully explored for the removal of antibiotics from aqueous environments using adsorption techniques [21,32].

In our previous work, we have presented the functionalization of styrene–divinylbenzene copolymers with N,N-dialkyl 2-hydroxyethyl ammonium groups [33,34], and with aminobenzoic acid groups [35], by polymer-analogous reactions.

In this article, we present, for the first time, the adsorption performances of macroporous styrene–divinylbenzene copolymers bearing p-aminobenzoic acid groups against antibiotics. The polymeric adsorbents from the present study are different from those used in our previous work [35].

The purpose of the present study was to obtain new polymeric adsorbents, starting from macroporous chloromethylated styrene–divinylbenzene copolymers, with different degrees of crosslinking (6.7% (PAB1), 10% (PAB2), and 15% (PAB3) divinylbenzene). These functionalized solid copolymer adsorbents are easily recovered from the rection medium by simple filtration, with no contamination of the environment. New polymeric adsorbents were characterized by a wide range of analytical methods: FTIR spectroscopy, thermogravimetric analysis, and EDX, SEM, and BET analysis. The polymeric adsorbents functionalized with p-aminobenzoic acid active groups (PAB1, PAB2 and PAB3) were tested by extensive kinetic and thermodynamic studies in the adsorption of antibiotics (tetracycline, sulfamethoxazole, and amoxicillin) from aqueous solutions. The best adsorption capacity was observed for tetracycline, while the adsorption of amoxicillin did not show positive results.

## 2. Results and Discussion

### 2.1. Adsorbent Synthesis and Characterization

**Scheme 1:**
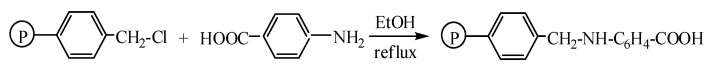
The functionalization reaction of the copolymers with aminobenzoic acid.

Copolymers chemically modified with pendant active groups containing nitrogen and/or oxygen were synthetized and characterized. The synthesis reaction is shown in Scheme 1.

**Figure 1:**
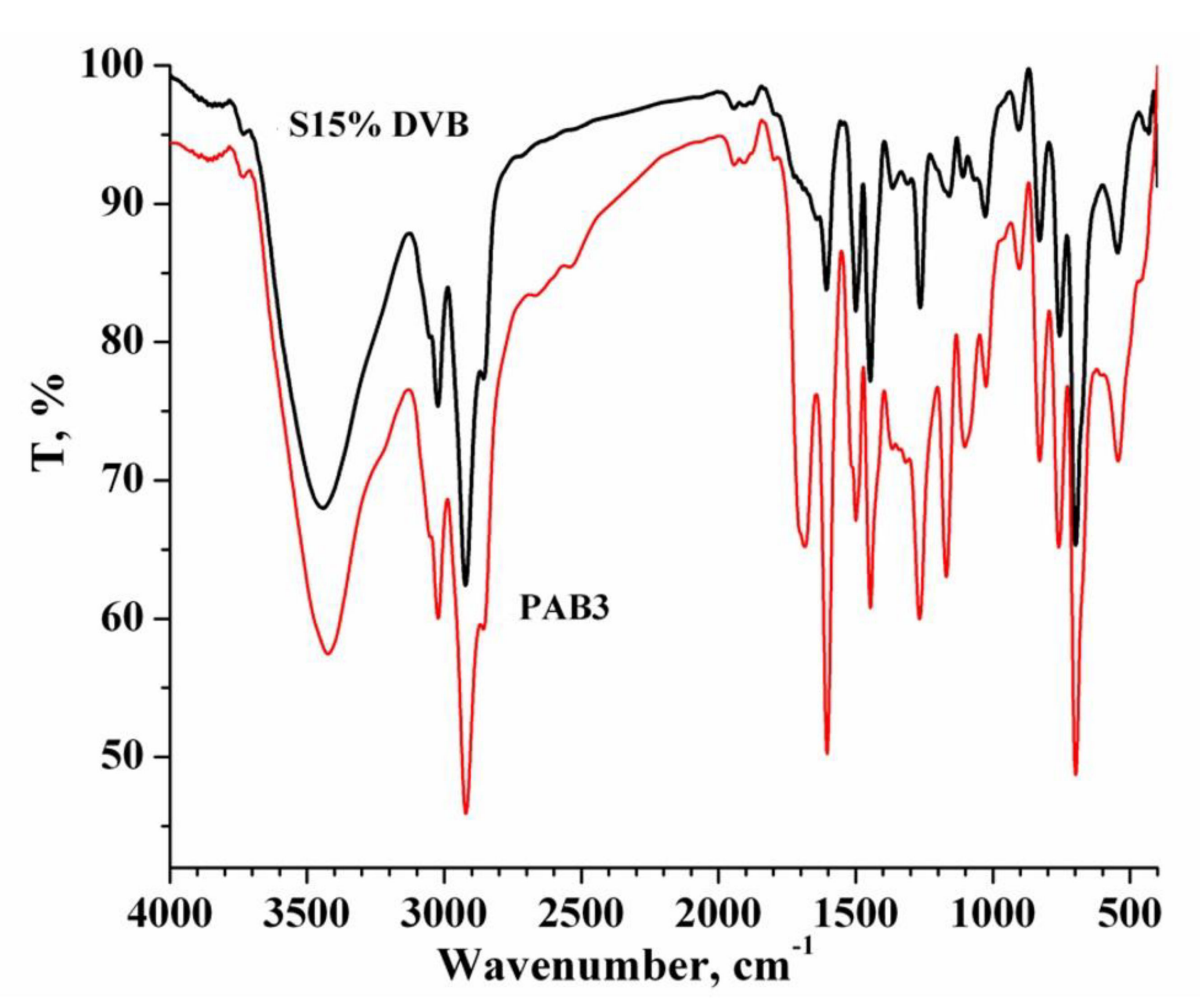
FTIR spectra of the source copolymer and functionalized copolymer PAB3.

The evolution of the functionalization reactions was observed by FTIR spectroscopy. The FTIR spectrum (see Figure 1) shows the bands corresponding to the source S–15%DVB chloromethylated copolymer and respectively the absorption bands of the functionalized product PAB3. A decrease in the intensity of the γCH_2_Cl band at 1260 cm^−1^, was observed simultaneously with the appearance of the C=O carboxylic band from 1685–1695 cm^−1^. The presence of COOH groups grafted on the copolymer was confirmed by the appearance of the C=O carboxylic band from 1748 cm^−1^, assigned to the C=O stretching vibrations in aryl-COOH [36]. The presence of both COOH and NH groups on the PAB3 copolymer was confirmed by the intense broad band at 3424 cm^−1^, which was attributed to the stretching vibration of both the OH and N-H groups. The spectrum showed an increase in the intensity of both the 1604 cm^−1^ band and the 1120 cm^−1^ band, which was attributed to the δNH and C–N stretching of aryl-NH-CH_2_- group [37]. The spectra of the products PAB1 and PAB2 are very similar due to the presence of the same type of copolymer matrix.

**Figure 2:**
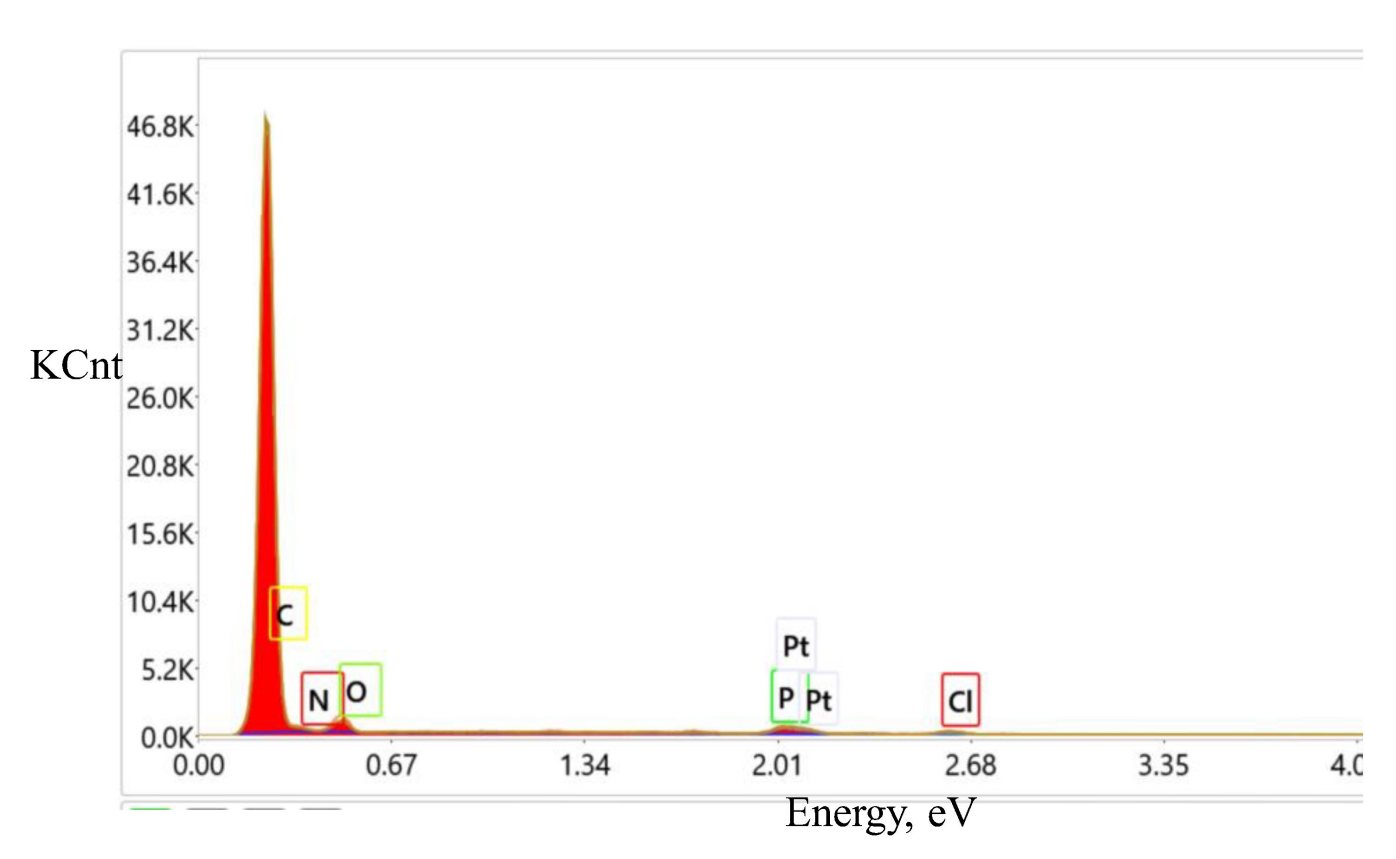
EDX spectrum of PAB2.

The energy dispersive X-ray analysis (EDX) for the adsorbent PAB2 is presented in Figure 2.

The data obtained from the X-ray energy dispersion, EDX, and analysis (see Table 1) of the three copolymer products functionalized with the aminobenzoic groups PAB1 (styrene—6.7%DVB), PAB2 (styrene—10%DVB), and PAB3 (styrene—15%DVB), confirmed the functionalization by the presence and content of nitrogen (PAB1: N% = 0.47; PAB2: N% = 0.85; PAB3: N% = 1.30) and oxygen (PAB1: O% = 7.61; PAB2: O% = 3.1; PAB3: O% = 10.95).

**Figure 3:**
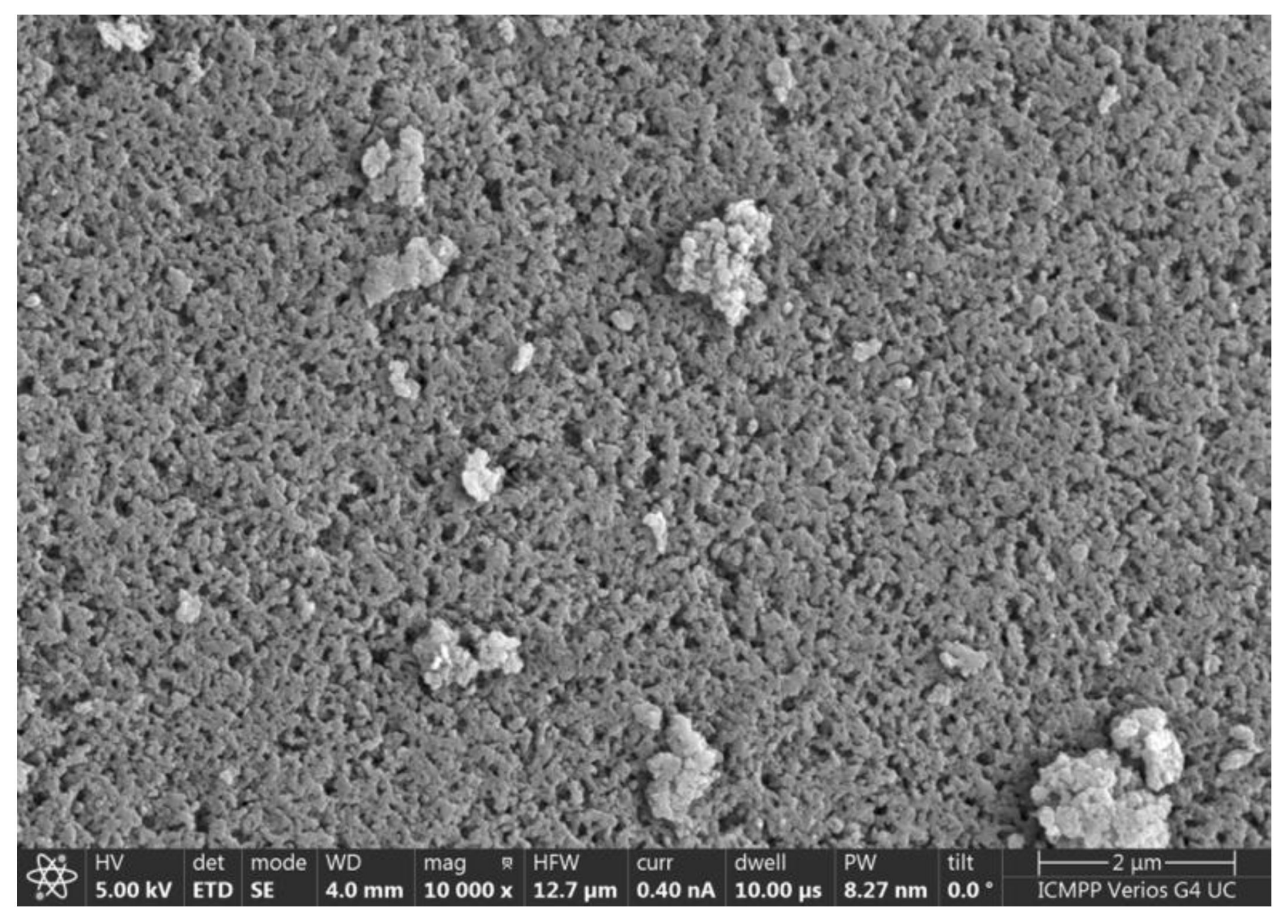
SEM image of PAB3 sample.

In the scanning electron microscopy (SEM) image of the PAB3 sample (see Figure 3), the macropores are visible among the agglomerations of the microspheres; the sample is characterized by a dense structure. Thus, the PAB3 sample can be considered a macroporous material, which consists of agglomerations of microspheres, between which a porous structure with a labyrinth of channels can be observed.

**Figure 4:**
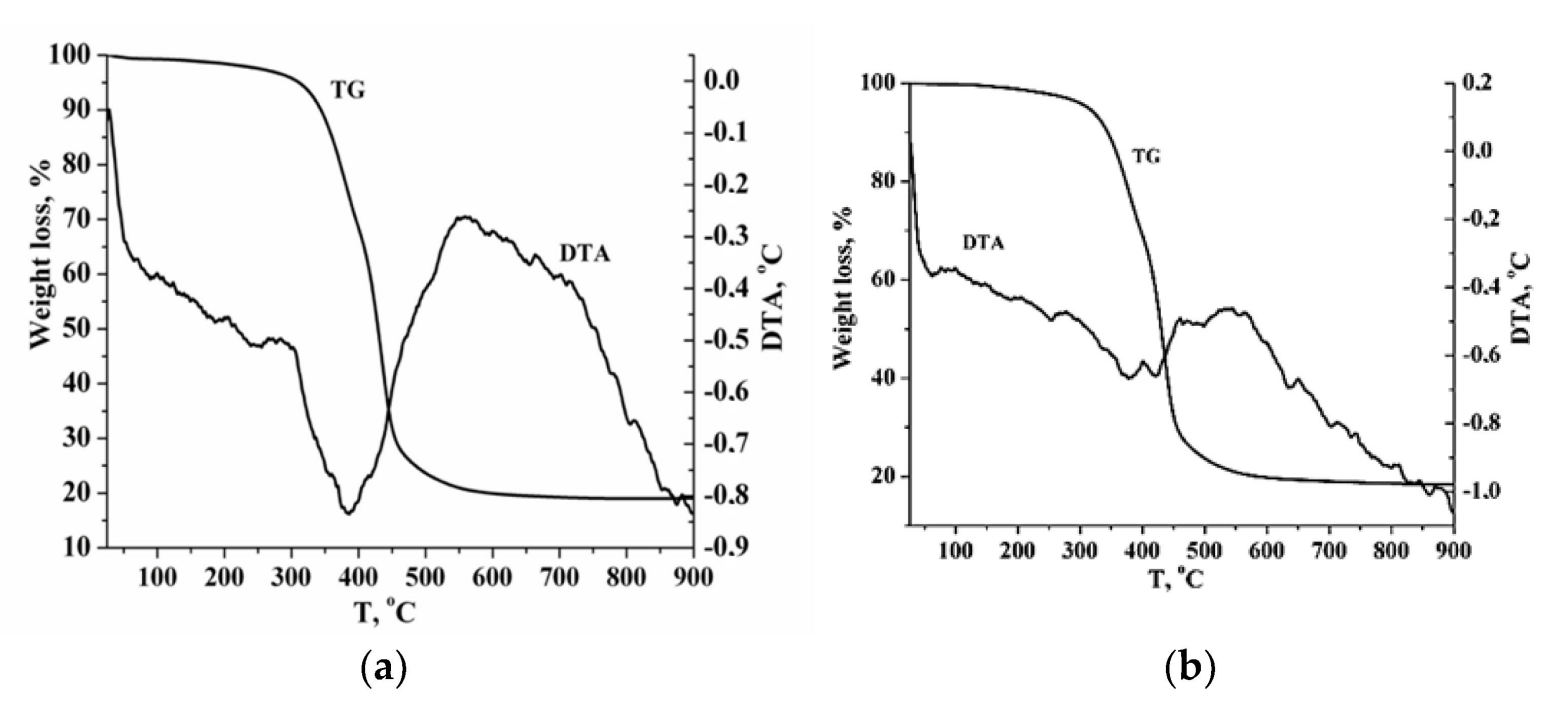
TG/DTA curves of PAB3 sample in: (**a**) nitrogen atmosphere, (**b**) air atmosphere.

The thermal stability of the macromolecular adsorbents was studied by TG and DTA analysis. Thermograms of the PAB3 adsorbent are presented in Figure 4.

The thermal behavior of the PAB3 sample in an atmosphere of nitrogen (Figure 4a) showed that weight loss began from 25 °C, which was due to the evaporation of physically adsorbed water or residual solvent (ethanol) in the adsorbent. Further, from ~150 °C, the PAB3 sample started to decompose. Weight loss was observed in the temperature domain of 300–450 °C due to the decomposition of the C-C, C-N, and C-O bonds of the pendant active groups of the aminobenzoic type. The process of the decomposition of the pendant groups took place at a high speed, at 396 °C and 450 °C. The DTA suggest that the compound underwent thermal and oxidative decomposition both in nitrogen and in air (Figure 4a). After the temperature range of ~400 °C to 600 °C, another decomposition process occurred; this was mainly due to the degradation of the copolymer chains, and the weight loss had only an exothermic peak. In this temperature domain, the peak was relatively large, which suggested that only oxidative decomposition occurred. The final residue at 900 °C was ~19%.

The thermal behavior of the PAB3 sample in air (Figure 4b) showed an endothermic effect in the range of 60–450 °C, with a first weight loss of about 30%, followed by a sequence of exothermic effects in the range of 450–800 °C, with a weight loss of about 47% due to the oxidative degradation of the PAB3 sample. The total weight loss of the PAB3 sample at 900 °C was of about 81%. The difference in the thermal behavior of the PAB3 in air (Figure 4b) and in nitrogen (Figure 4a) was suggested by the more accentuated endo and exothermic processes in the air atmosphere.

The products PAB1, PAB2, and PAB3 were very stable in the range of temperatures intended for their use as adsorbents (up to ~150 °C).

**Figure 5:**
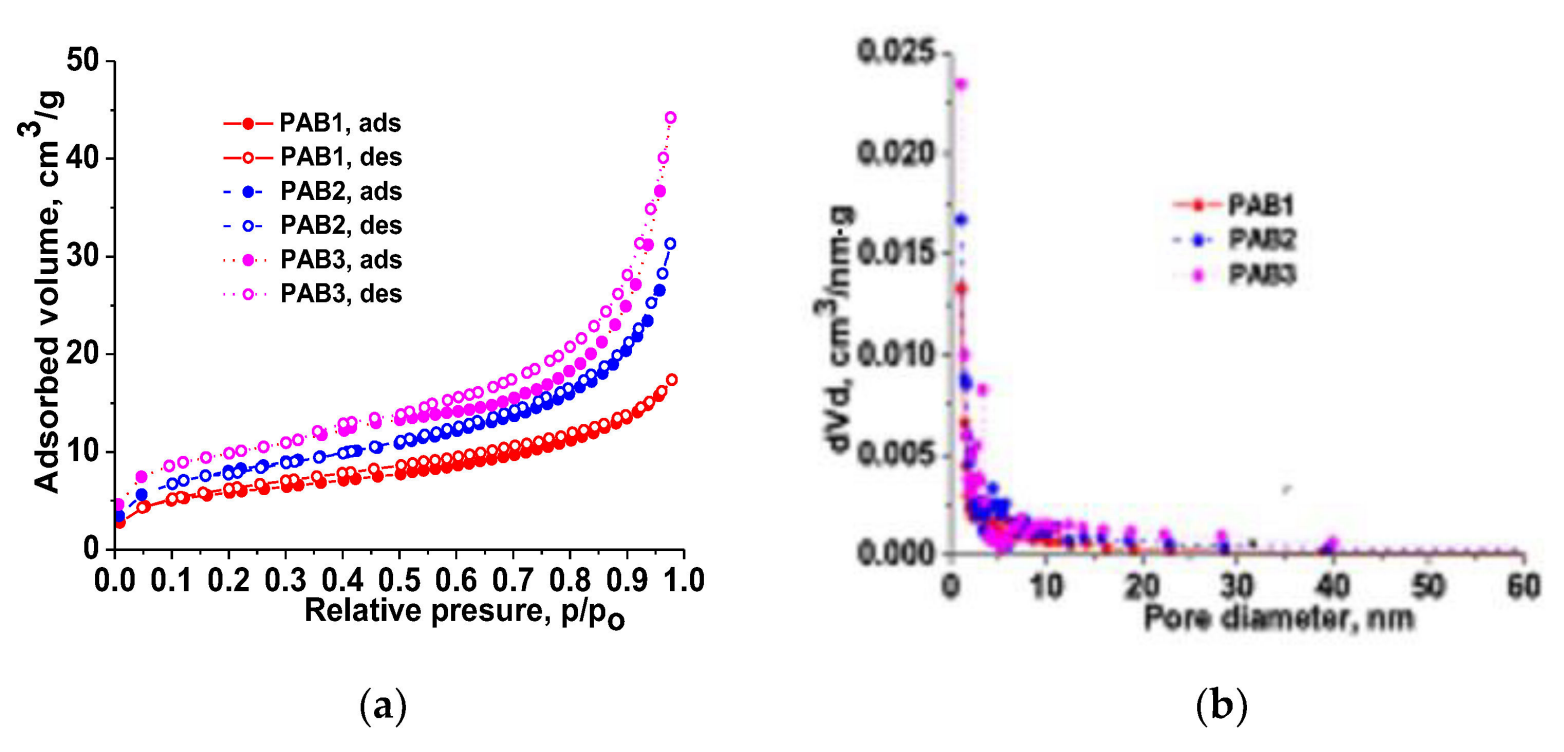
BET isotherms (**a**) and BJH distribution of pore diameters (**b**) for the PAB sorbents.

The adsorption of organic compounds is strongly influenced by the morphology and textural properties of the adsorbent: the distribution of surface functional groups, porosity, specific surface area, pore dimensions, and total pore volume [38,39]. The BET isotherms and BJH distribution of the pore diameters for the PAB1, PAB2, and PAB3 are compared in Figure 5.

As can be observed in Figure 5a, all the isotherms corresponded to type IV, as categorized by the IUPAC classification, with respect to a hysteresis loop, indicating the uniformity and regularity of the textural properties of all the sorbents. The textural parameters calculated based on the obtained isotherms for the PAB1, PAB2, and PAB3 are summarized in Table 2.

As can be seen in Table 2, the BET surface areas of the PAB adsorbents, which were calculated by applying the BET equation to the linear part (0.05 < P/P_0_ < 0.305) of the adsorption isotherms, were 20.14, 27.89, and 34.56 m^2^/g, for PAB1, PAB2, and PAB3, respectively. These values show that the specific surface areas and total pore volumes of the porous sorbents increased from PAB1 to PAB3, which was in good agreement with the increase in the DVB content in the pristine S-DVB matrix.

### 2.2. Adsorption Characterization

In the graphical representations, we mostly present the data for the best-performing adsorbent, PAB3.

The adsorption capacity onto the polymeric adsorbents was calculated using Equation (1):

(1) $$q_{e}=\frac{V\left(C_{i}-C_{e}\right)}{m}$$

where *V* is the volume of solution (L), m is the mass of the dry adsorbent (g), and *C_i_* and *C_e_* (mM/L) are the initial and equilibrium concentrations of the sulfamethoxazole and tetracycline, respectively.

**Figure 6:**
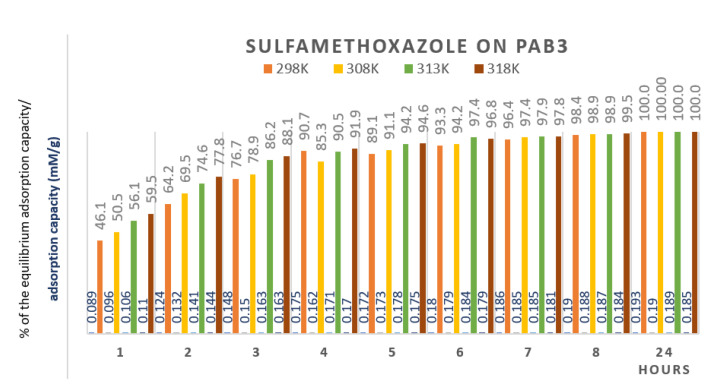
Evolution of the % of the equilibrium adsorption capacity and adsorption capacity versus time for the adsorption of sulfamethoxazole on the adsorbent PAB3.

**Figure 7:**
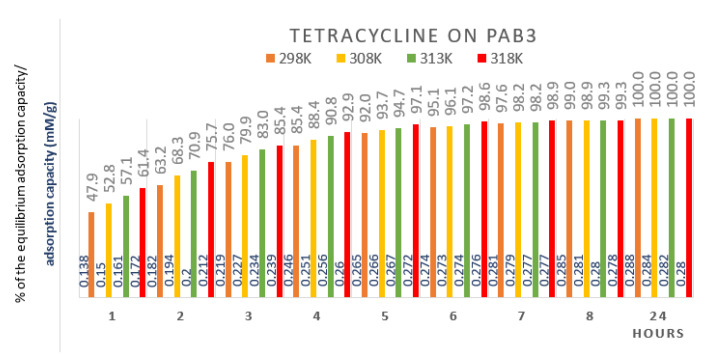
Evolution of the % of the equilibrium adsorption capacity and adsorption capacity versus time for the adsorption of tetracycline on the adsorbent PAB3.

The comparison of the evolution with the time of the adsorption process on the adsorbent PAB3 is presented in Figure 6 and Figure 7.

As can be seen from Figure 6 and Figure 7, the adsorption process was fast: at least 80% of the maximum adsorption capacity (equilibrium adsorption capacity) was attained in 4 hrs. Similar behavior was observed with the adsorbents PAB1 and PAB2.

**Figure 8:**
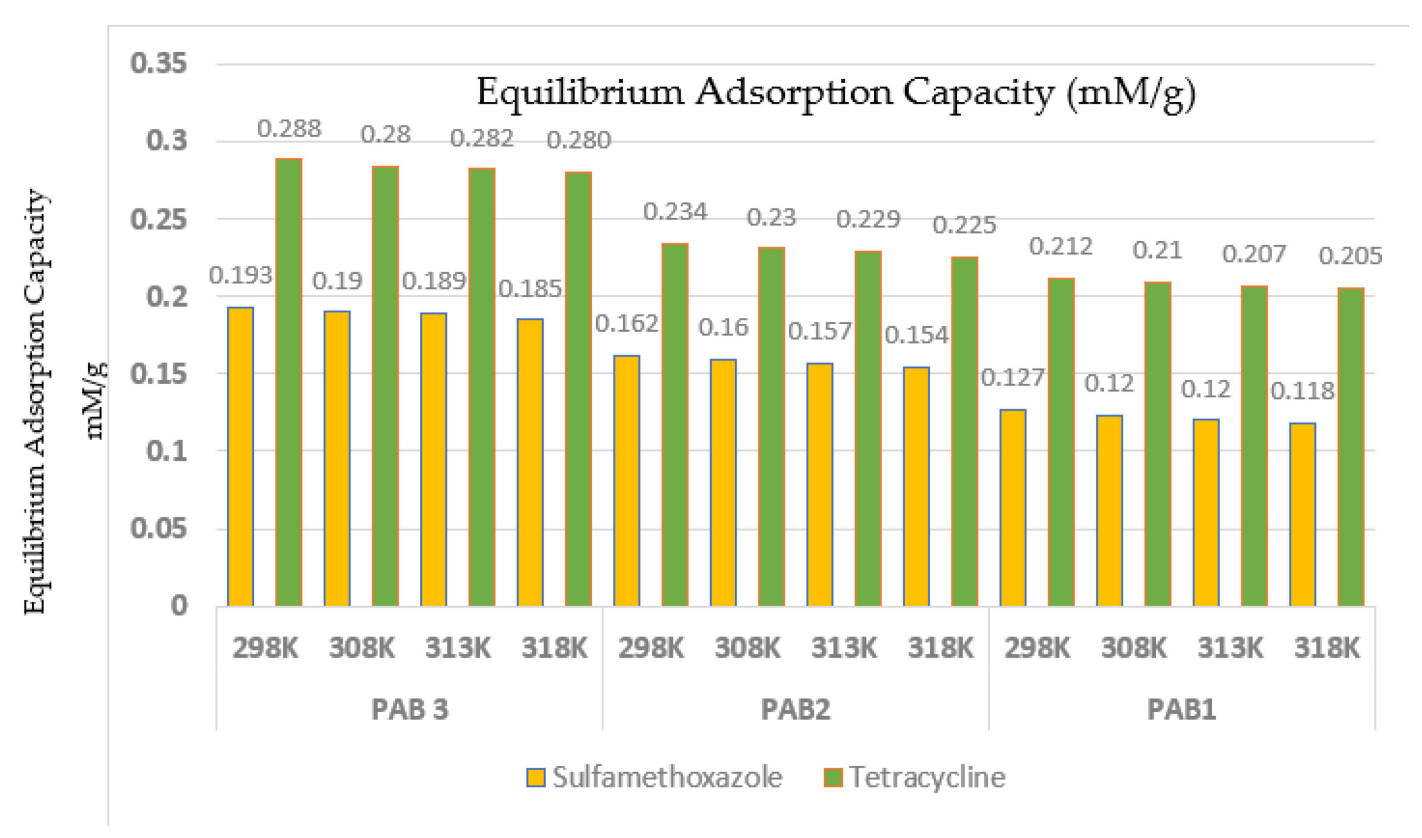
Equilibrium adsorption capacity on the studied adsorbents for an initial adsorbate concentration of 3 mM/L.

A comparison of the performances of the adsorbents PAB1, PAB2, and PAB3 in the adsorption of the sulfamethoxazole and tetracycline, respectively, at four temperatures (298 K, 308 K, 313 K and 318 K), for an initial concentration of adsorbate solution of 3 mM/L, is presented in Figure 8.

As can be seen, all the adsorbents under study were effective in the adsorption of the sulfamethoxazole and tetracycline. The performances were ranked in the following order:

PAB1 < PAB2 < PAB3

**Figure 9:**
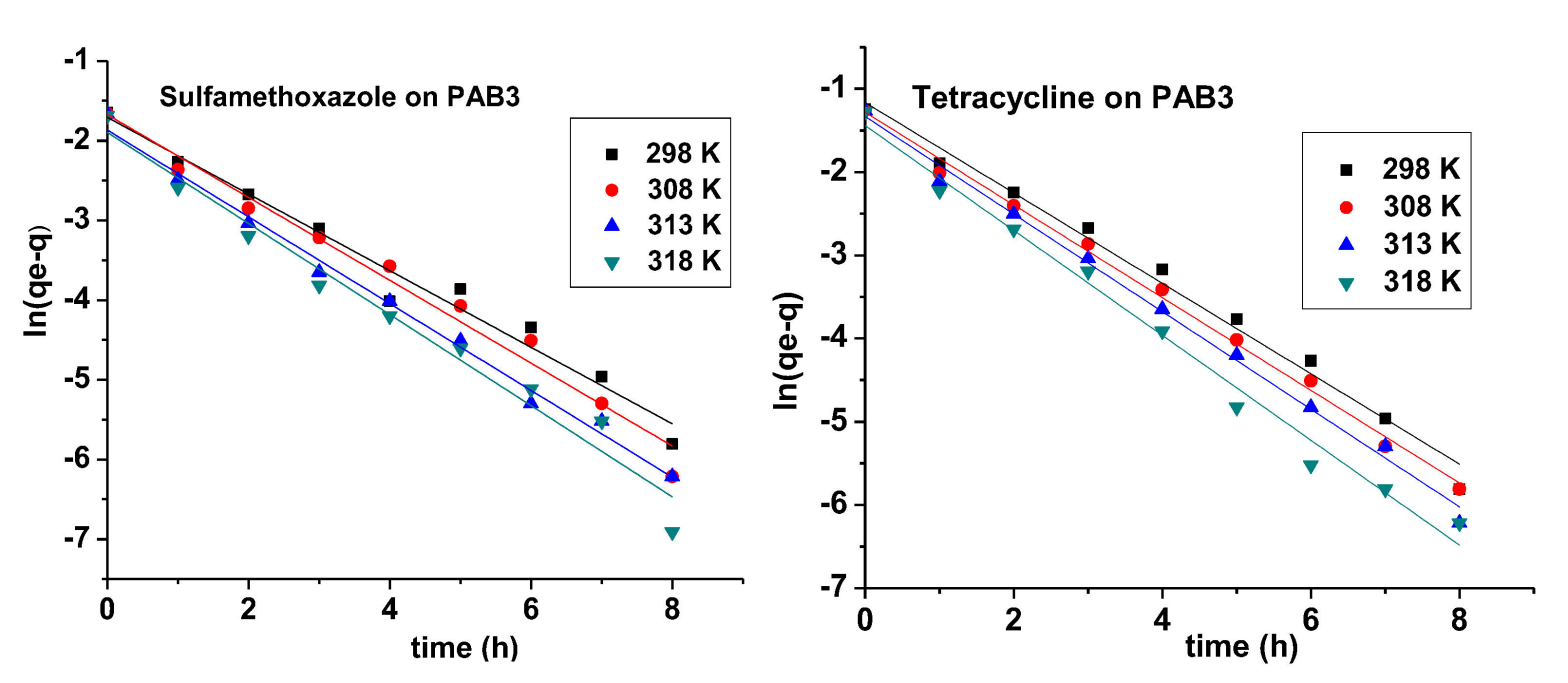
Plots of Lagergren pseudo-first-order kinetic equation for the adsorption of sulfamethoxazole and tetracycline, respectively, on PAB3 polymeric adsorbent.

The performance of the adsorbents can be correlated with the morphology and textural properties of the functionalized copolymers. The best performance was observed with the macroporous adsorbent PAB3, which had the largest surface area, total pore volume, and mean pore volume (see Table 2). The equilibrium adsorption capacity was also dependent on the chemical nature of the adsorbate, with the tetracycline being better adsorbed in comparison with the sulfamethoxazole. As can be observed in Figure 9, increasing the operation temperature had a rather minor effect, which suggests that the adsorption process was most likely thermodynamically controlled and not kinetically controlled. The slight decrease in the equilibrium adsorption capacity versus the temperature indicates that the adsorption process was most likely exothermic.

### 2.3. Kinetic Studies

Kinetic adsorption experiments were carried out in order to obtain information about the mechanism of adsorption at the solid–liquid interface. In this study, the Lagergren [40] pseudo-first-order kinetic model (PFO) (Equation (2)) and the pseudo-second-order Ho and McKay (PSO) [41,42,43,44,45,46] kinetic model (Equation (3)) were used to test the experimental data.

The PFO model is based on the assumption that the rate of adsorbate uptake with time is directly proportional to the difference between the equilibrium concentration of the solution and the amount of adsorbate on the solid adsorbent with time, an assumption that is generally true in the initial stages of the adsorption process. It is commonly observed that the kinetics follow the PFO model when the adsorption process is diffusion-controlled.

(2) $$\ln\left(q_{e}-q\right)=\ln q_{e}-k_{1}t$$

where *q* and *q_e_* represent the adsorption capacity at time *t* and at equilibrium, respectively, in mM/g, and *k_1_* is the adsorption rate constant, in min^−1^.

The PSO kinetic model is based on the assumption that the rate-limiting step is chemosorption and predicts the behavior over the whole range of adsorption. If this assumption is true, the Ho–McKay model presumes that the adsorption rate is dependent on the adsorption capacity, and not on the concentration of adsorbate in the solution.

(3) $$\frac{t}{q}=\frac{1}{k_{2}q_{e}^{2}}+\frac{1}{q_{e}}t$$

where *k_2_* is the PSO rate constant, g mg^−1^ min^−1^.

**Figure 10:**
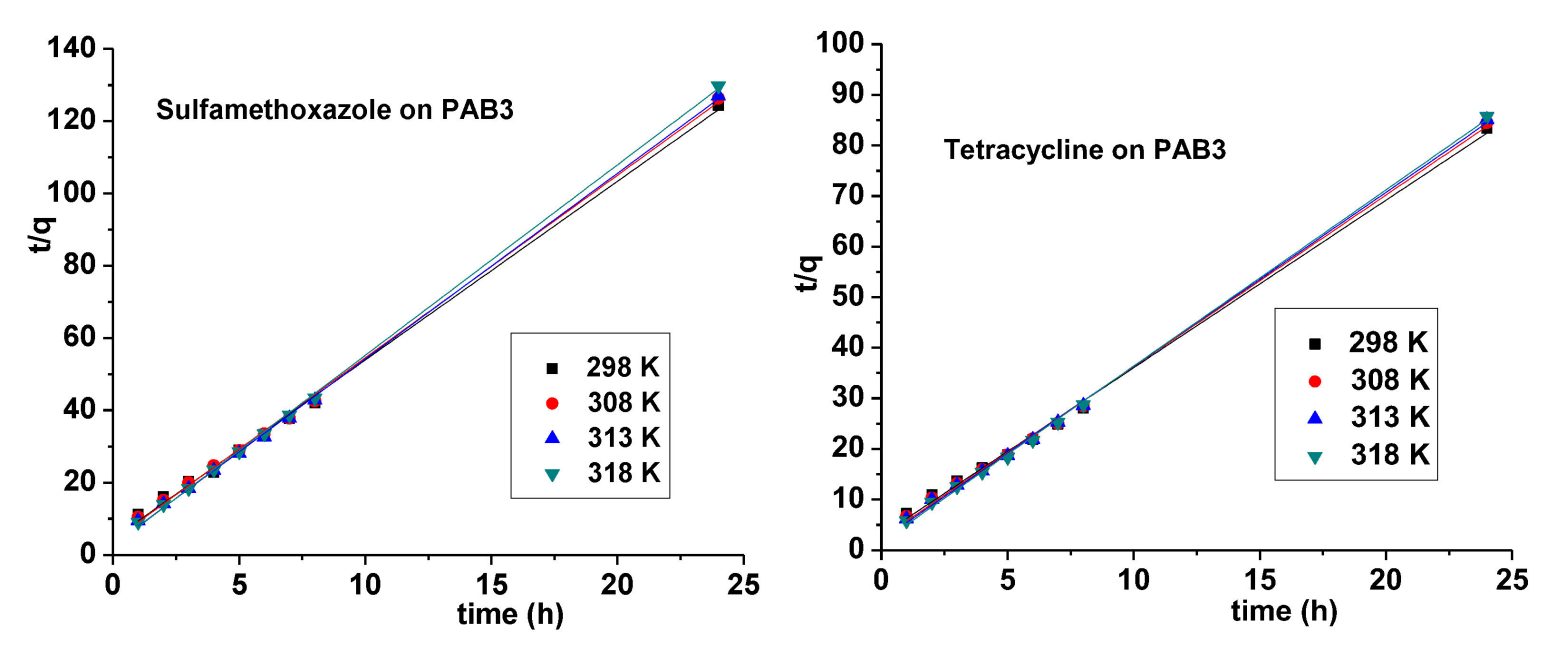
Plot of Ho–McKay pseudo-second order kinetic equation for the adsorption of sulfamethoxazole and tetracycline, respectively, on PAB3 polymeric adsorbent.

Typical graphic representations of the two kinetic models are presented in Figure 9 for the Lagergren model and in Figure 10 for the Ho–McKay model, for the adsorption on the adsorbent PAB3. The slopes and intercepts of the plots of *ln(qe-q)* vs. *t* were used to determine the PFO rate constants, *k_1_*. The PSO rate constants, *k_2_*, were estimated from the intercept and slope of *t/q* vs. *t*. The values of the rate constants and the determination coefficients (*R^2^*) obtained in all the cases are shown in Table S1.

The data presented in Table S1 show that the determination coefficients found for the PFO kinetic model were much lower than those obtained for the PSO model. This proves that the kinetics of sulfamethoxazole and tetracycline removal by adsorption on the studied polymeric adsorbents are best described by a PSO kinetic model.

### 2.4. Equilibrium Studies—Adsorption Isotherms

In this paper, we studied the adsorption of sulfamethoxazole and tetracycline from aqueous solutions using two-parameter isotherms, Langmuir and Freundlich, and three-parameter isotherms, Sips and Redlich–Peterson. For precise calculations of the isotherms characteristic parameters we have used the non-linear form of the isotherms (in order to avoid inherent errors due to linearization) and non-linear fitting of the experimental equilibrium data.

The *Langmuir* isotherm [47], although initially introduced to describe the adsorption of the gases on the solid adsorbents, was subsequently used extensively for the adsorption at the liquid–solid interfaces. The isotherm assumes that the adsorption surface is homogeneous, with active adsorption centers that are identical and uniformly distributed, and with no interaction between the vicinal centers. Each adsorption center can accommodate a single adsorbate molecule. The model considers that the adsorption rate is proportional to the free fraction of the adsorbent and that the desorption rate is proportional to the fraction of the adsorbent occupied. At equilibrium, the two rates become equal. The non-linear form of the *Langmuir* isotherm is presented in Equation (4):

(4) $$q_{e}=q_{\max}\frac{K_{L}\cdot C_{e}}{1+K_{L}\cdot C_{e}}$$

where *q_e_* is the equilibrium adsorption capacity of the mM·g^−1^ adsorbent, *C_e_* is the equilibrium molar concentration of the adsorbate in the solution, mM·L^−1^, *K_L_* is the Langmuir equilibrium constant L·mM^−1^, and *q_max_* is the maximum adsorption capacity of the mM g^−1^ adsorbent.

The essential features of the Langmuir isotherm can be expressed in terms of the dimensionless constant *R_L_*, the separation factor:

(5) $$R_{L}=\frac{1}{1+K_{L}\cdot C_{0}}$$

Based on the values of the separation factor, the adsorption is considered *unfavorable* if *R_L_* > 1, *linear* if *R_L_* = 1, *favorable* if 0 < *R_L_* < 1, and *irreversible* if *R_L_* = 0.

The *Freundlich* isotherm [48] is an empirical adsorption isotherm that is used in adsorption studies on solid adsorbents with heterogenous surfaces and at medium concentrations of adsorbate solutions:

(6) $$q_{e}=K_{F}\cdot C_{e}^{\frac{1}{n}}$$

where *q_e_* is the equilibrium adsorption capacity, mM·g^−1^, *C_e_* is the equilibrium molar concentration of the adsorbate in solution, mM·L^−1^, *K_F_* is the Freundlich constant, mM^(1−1/n)^·L^1/n^·g^−1^, and *1/n* is the constant, depending on the nature and intensity of the adsorption interactions and reflecting the heterogenity degree of the adsorbent surface.

The Langmuir and Freundlich isotherms are in fact the two limit adsorption types at the solid–liquid interface. In order to better fit the experimental equilibrium data, three-parameter isotherms were used in order to correct for the inaccuracies eventually induced by the isotherms’ linearization.

The *Sips* adsorption isotherm [49], or the combined Langmuir–Freundlich isotherm, is also a three-parameter isotherm assuming cooperative sorbate–sorbate interactions. The model is suitable for describing adsorption on surfaces with a certain degree of heterogeneity, correcting for the limitation at the high adsorbate concentrations usually associated with the Freundlich model:

(7) $$q_{e}=q_{m}\frac{{\left(K_{S}\cdot C_{e}\right)}^{n}}{1+{\left(K_{S}\cdot C_{e}\right)}^{n}}$$

where *q_e_* is the equilibrium adsorption capacity, mM·g^−1^, *C_e_* is the equilibrium molar concentration of the adsorbate in solution, mM·L^−1^, *K_S_* is the Sips isotherm constant, L·mM^−1^, *n* is the heterogeneity parameter, with values between 0 and 1, and *q_m_* is the maximum adsorption capacity, mM g^−1^.

The *Redlich–Peterson* adsorption isotherm [50] is also a three-parameter isotherm, which takes into consideration a hybrid adsorption mechanism:

(8) $$q_{e}=\frac{K_{RP}\cdot C_{e}}{1+a\cdot C_{e}^{b}}$$

where *q_e_* is the equilibrium adsorption capacity, mM·g^−1^, *C_e_* is the equilibrium molar concentration of the adsorbate in solution, mM·L^−1^, *K_RP_* is the Redlich–Peterson isotherm constant, L·mM^−1^, *a* is the constant depending, on the nature of adsorption interactions, L· mM^−1^, and *b* is the exponent, with values between 0 and 1.

The experimental equilibrium data at four initial adsorbate concentrations (3.0, 2.5, 2.0, and 1.0 mM/L) and at four temperatures (298 K, 308 K, 313 K, and 318 K) are presented in Table S2.

The parameters of the adsorption isotherms were evaluated by non-linear regression analysis using the equilibrium data (see Table S2).

Due to the repetitive nature of the calculations, the non-linear regression analysis graphs of the adsorbates on the best-performing adsorbent, PAB3, are presented in the following figures. Similar regression data were also obtained for the adsorbents PAB2 and PAB1.

**Figure 11:**
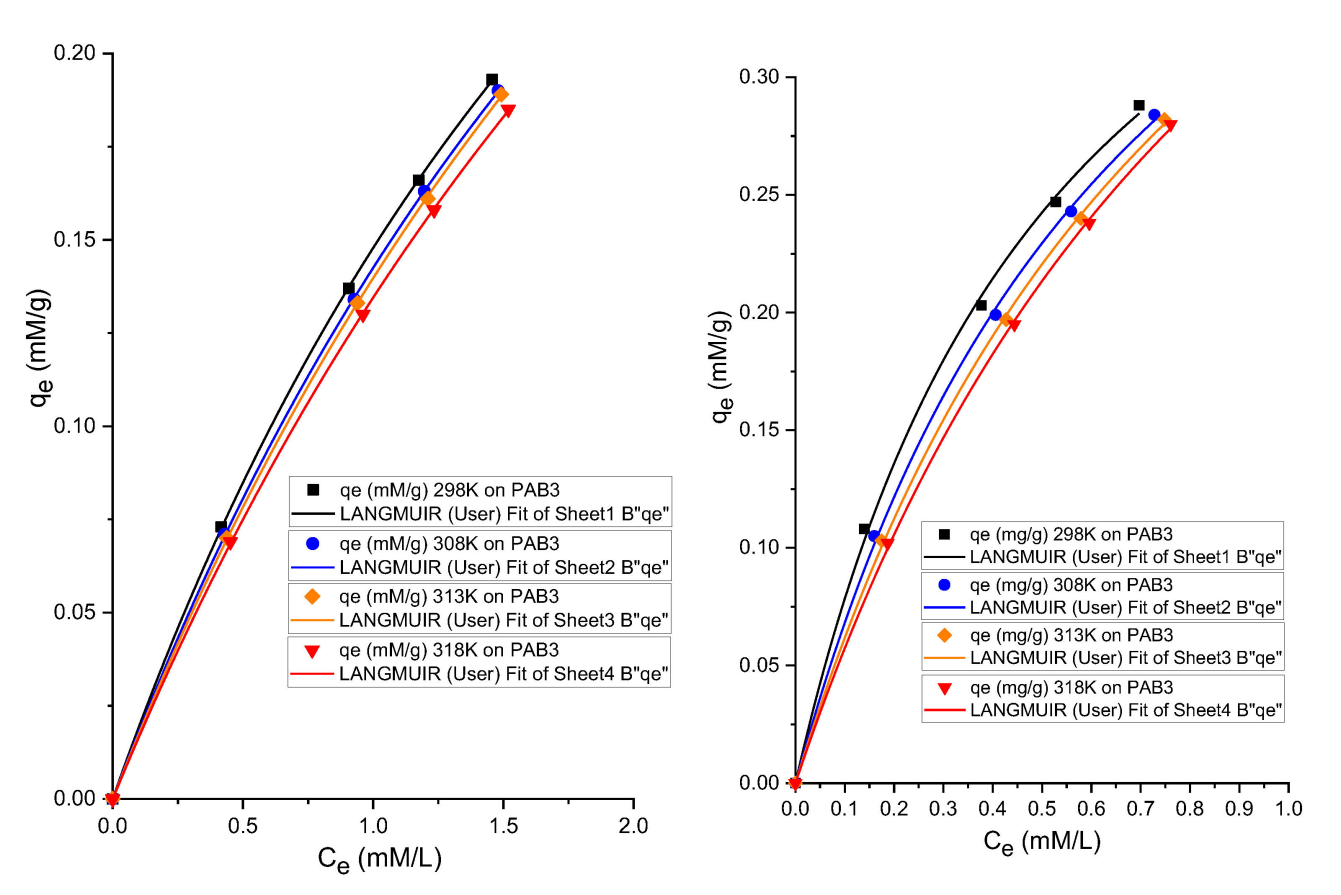
*Langmuir* isotherm non-linear regression analysis. Left: Sulfamethoxazole on PAB3. Right: Tetracycline on PAB3.

**Figure 12:**
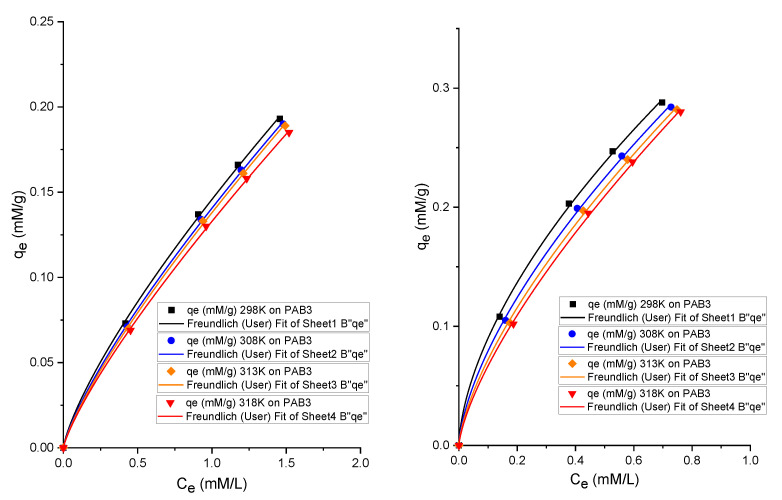
*Freundlich* isotherm non-linear regression analysis. Left: Sulfamethoxazole on PAB3. Right: Tetracycline on PAB3.

**Figure 13:**
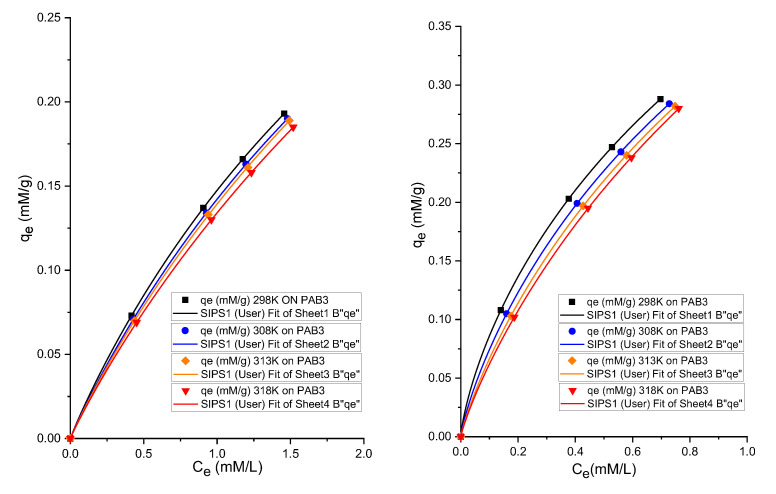
*Sips* isotherm non-linear regression analysis. Left: Sulfamethoxazole on PAB3. Right: Tetracycline on PAB3.

**Figure 14:**
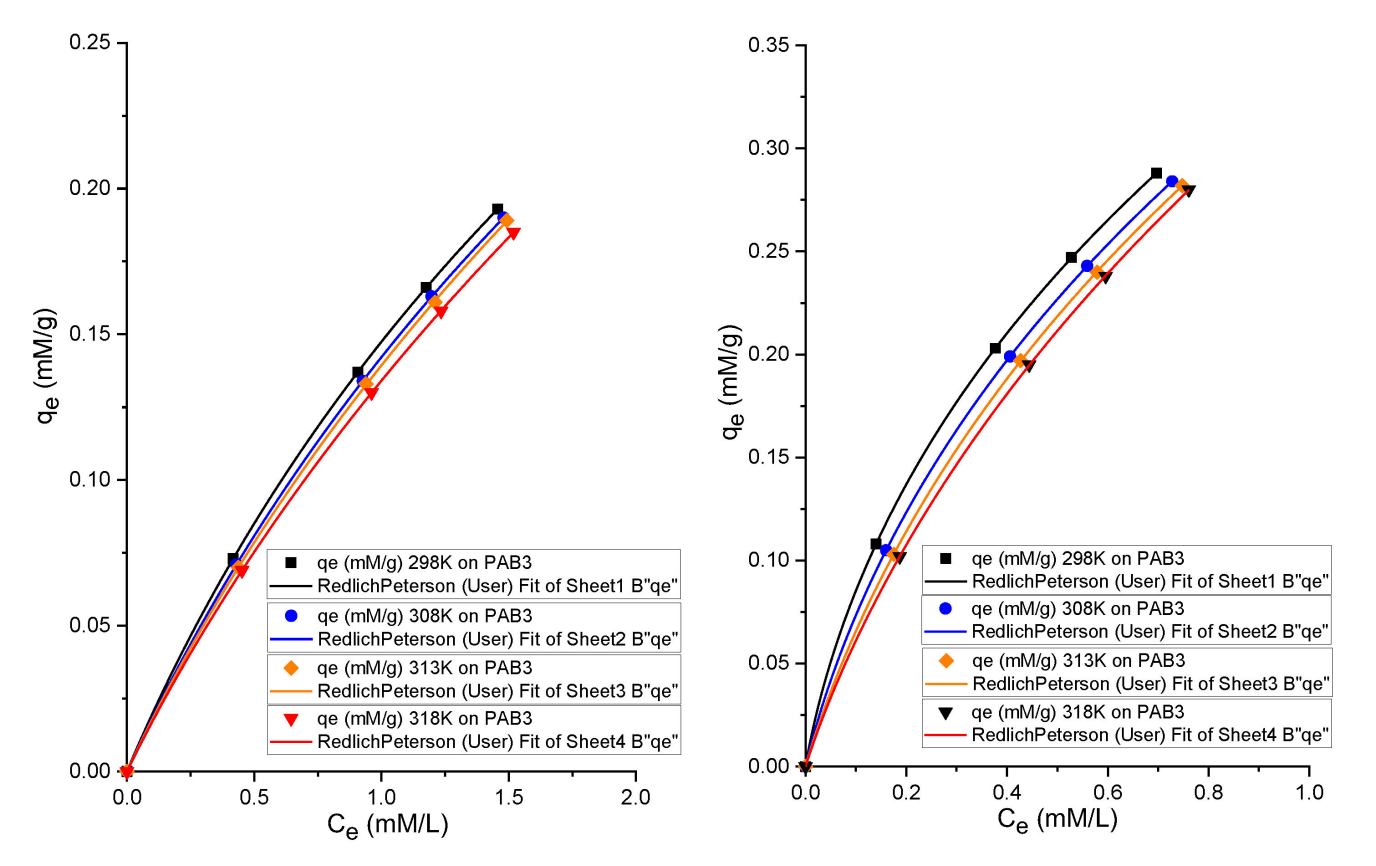
*Redlich–Peterson* isotherm non-linear regression analysis. Left: Sulfamethoxazole on PAB3. Right: Tetracycline on PAB3.

The data for the non-linear regression analysis using the Langmuir, Freundlich, Sips, and Redlich–Petersen adsorption isotherms are presented in Figure 11, Figure 12, Figure 13 and Figure 14 for the adsorption of the studied adsorbates on the best-performing adsorbent, PAB3. Similar data were also obtained from the study of the adsorption on the adsorbents PAB1 and PAB2 and are not presented in detail.

The isotherm parameters calculated for the adsorption of the sulfamethoxazole are presented in Table S3 and those for the adsorption of the tetracycline in Table S5.

The data obtained by the non-linear regression analysis indicated an excellent fit with the experimental equilibrium data. The best correlations were obtained with the Sips and Redlich–Peterson adsorption isotherms (see the values of the coefficients of determination, R-Squared, *R^2^*). The Sips and Freundlich isotherms indicated a rather high degree of heterogeneity of the adsorbent surface. These findings suggest that the adsorption mechanism is most likely hybrid.

The calculated value of the separation factors *R_L_* (see Table 3 and Supplementary Materials Table S4) clearly indicate that the adsorption process is *favorable* for all the adsorbents studied: 0 < *R_L_* < 1.

In the absence of specific surface functional groups that can determine the interactions between an adsorbent and adsorbate molecules, adsorption over porous adsorbents primarily depends on the surface area and porosity of the material [51]. The interactions between functionalized S-DVB adsorbents and pharmaceuticals, including antibiotics such as sulfamethoxazole and tetracycline, in aqueous solutions, can be attributed to various interactions [5,6,7,52]. Thus, based on the kinetic and thermodynamic studies, we presumed that the adsorption mechanism could involve electrostatic interactions, π–π interaction (stacking), hydrophobic interactions, and/or hydrogen bonding. Based on the nature of the active centers of the adsorbents (the presence of the secondary amine group and of the carboxyl group) and the chemical structure of the antibiotics (Figure S1), the cooperative interactions between the functional groups of the adsorbent and the antibiotics could explain why the tetracycline was better-adsorbed than the sulfamethoxazole.

## 3. Experimental Section

### 3.1. Materials and Methods

The macroporous chloromethylated styrene–divinylbenzene copolymers S—6.7%DVB (supplied by Purolite, Victoria, Romania, %Cl = 14.22), S—10%DVB (%Cl = 10.25), and S—15%DVB (%Cl = 11) (supplied by “Petru Poni” Institute of Macromolecular Chemistry, Iasi, Romania) [34,35], p-aminobenzoic acid (Reactivul, Bucharesct, Romania, p.a.), and ethanol (Chimopar, Bucharest, Romania, p.a.) were used for preparation of functionalized polymeric adsorbents with p-aminobenzoic acid groups (6.7% (PAB1), 10% (PAB2), and 15% (PAB3)).

Polymeric materials were characterized by the FTIR spectra (KBr pellets) on a JASCO FTIR spectrophotometer (JASCO, Tokyo, Japan) in the range of 4000–400 cm^−1^ and maximum resolution of FTIR spectra of 0.4 cm^−1^.

The thermal properties of the new polymeric products (PAB1, PAB2, PAB3) were characterized through thermogravimetric analysis on a TGA/SDTA 851-LF1100 Mettler machine (Greifensee, Switzerland) at a heating rate of 10 °C/min under a nitrogen/air atmosphere and temperature range from 25 to 900 °C.

The energy dispersive X-ray analysis (EDX) and study the surface morphology of the functionalized copolymers were performed using a Quanta FEG 250 microscope—at an accelerating voltage of 20 kV—(FEI Company, Eindhoven, the Netherlands).

The specific surface area (S_sp_) of the polymeric materials and the pore size distribution were estimated from N_2_ adsorption–desorption experiments conducted at 77 K using an Autosorb-1-MP surface-area analyzer (Quantachrome Company, Boynton beach, FL, USA). Before measurements, the sample was outgassed at room temperature, for two hours. The isotherms of nitrogen adsorption/desorption were measured at values of the relative pressure p/p_0_ in the domain of 0.01–1.0. The S_sp_ was estimated from the linear portion of the adsorption isotherms (0.05–0.35) by Brunauer–Emmett–Teller (BET) method. The Barrett–Joyner–Halenda (BJH) theory was used to estimate the pore size distribution from the adsorbed volume of nitrogen determined at saturation at values of the relative pressure p/p_0_ nearest to unit (0.95).

### 3.2. Synthesis of Chemically Modified Copolymers with p-Aminobenzoic Groups

A reaction procedure described in our previous works [36,37] for obtaining p-aminobenzoic acid functionalized onto chloromethylated styrene–divinylbenzene copolymers was used in this work.

The chemically modified copolymers (see Scheme 1) were obtained using a molar ratio of chloromethyl groups to p-amino-benzoic acid of 1:1, working in ethyl alcohol, at the solvent reflux temperature. The reaction products were separated by filtration, washed, and dried at 50 °C for 24 h.

### 3.3. Adsorption Experiments

The pharmaceutical Sumetrolim^®^ tablet, manufactured by Egis Pharmaceuticals PLC, Hungary, contained 400 mg of sulfamethoxazole. An accurately weighed 0.753 g sulfametoxazole tablet was transferred to 1000 mL volumetric flask and dissolved, to achieve a concentration of 3 mM/L. The solution was then diluted to obtain different solutions of sulfamethoxazole with the required initial concentrations (2.5 mM/L, 2 mM/L, and 1 mM/L, respectively).

Tetracycline was purchased from Arena Group SA, Romania. A total of 1332 g of tetracycline was dissolved in 1 L of deionized water to prepare the tetracycline solution with an initial concentration of 3 mM/L, and was then diluted to prepare additional solutions with the required tetracycline concentrations (2.5 mM/L, 2 mM/L, and 1 mM/L, respectively).

The influence of different parameters on adsorption of sulfamethoxazole and tetracycline onto the three synthesized polymeric adsorbents was studied. To study the effect of the contact time, initial concentration, and temperature on adsorption, the experiments were carried out with samples of 0.2 g adsorbent in 25 mL of 3 mM/L, 2.5 mM/L, 2 mM/L, and 1 mM/L sulfamethoxazole and tetracycline solution, respectively. The suspensions were shaken at 180 rpm for 24 h using a Julabo SW22 shaker (JULABO GmbH, Selma Bach, Germany), at four different temperatures (298 K, 308 K, 313 K, and 318 K).

The solutions were tested hourly for the first 8 h. Every hour, a sample of 2 mL of solution was analyzed to determine the antibiotic compound uptake, using UV spectrophotometry. To determine the residual concentrations of antibiotics, we used a Shimadzu UV mini 1240 UV-VIS spectrophotometer (Shimadzu, Duisburg, Germany). The measurements were conducted at wavelengths of 257 nm for sulfamethoxazole solutions and 357 nm for tetracycline solutions, respectively.

## 4. Conclusions

In this article, we obtained styrene–divinylbenzene copolymers functionalized with aminobenzoic acid groups by polymer-analogous reactions. The obtained products, PAB1, PAB2, and PAB3, were characterized by a wide range of methods: FTIR, thermogravimetric analysis, and EDX, SEM, BET, and BJH analysis, and were tested by extensive kinetic and thermodynamic studies on the adsorption of antibiotics (tetracycline, sulfamethoxazole, and amoxicillin) from aqueous solutions.

From the data presented in our article, a number of conclusions can be drawn regarding the behaviors of the functionalized styrene–divinylbenzene copolymers as adsorbents:

It was found that all the polymeric adsorbents modified by functionalization with aminobenzoic acid groups could be used in the adsorption processes of some antibiotics from aqueous solutions. They were tested in the removal of the sulfamethoxazole and tetracycline. Amoxicillin adsorption was also attempted, but it did not produce in positive results.

The adsorption capacity of the tested polymer adsorbents depended on the degree of crosslinking and the percentage of divinylbenzene in their structure, which affected the porosity, total surface area, pore volume, and pore distribution. The efficiency of the polymer adsorbents was found to increase in the following order: PAB1 < PAB2 < PAB3. This was in agreement with the increase in the cross-linking degrees and porosity.

The most effective adsorbent behavior for the removal of antibiotics was demonstrated by the PAB3 polymer adsorbent.

The adsorption capacity of the functionalized polymeric matrices depended on the nature and structure of the adsorbed antibiotic. The sulfamethoxazole and tetracycline were well adsorbed on all three adsorbents. In ascending order of adsorption capacity, antibiotics are placed in the sequence: sulfamethoxazole < tetracycline.

The adsorption process was rather fast: in the first 3–4 h, at least 80% of the maximum adsorption capacity was reached (see Figure 6, Figure 7 and Figure 8).

The kinetic studies of the sulfamethoxazole and tetracycline removal by adsorption on the studied polymeric adsorbents indicated that the process is best described by a pseudo-second-order kinetic model (Ho–McKay).

The increase in the operational temperature had a rather minor effect, which suggests that the adsorption process was most likely thermodynamically controlled and not kinetically controlled. The slight decrease in the equilibrium adsorption capacity versus the temperature indicated that the adsorption process was most likely exothermic.

The analysis of the adsorption isotherms indicated that the solid adsorbents had rather heterogeneous surfaces and suggested a probable hybrid adsorption mechanism and a *favorable* adsorption process.

## Figures and Tables

**Table 1:** X-ray energy dispersion, EDX, quantification of the PAB1, PAB2, and PAB3 samples.

Sample	wt %			
	C	N	O	Cl
PAB1	90.64	0.47	7.61	1.28
PAB2	91.40	0.85	3.10	1.43
PAB3	86.22	1.30	10.95	1.53

**Table 2:** BET analysis of styrene–divinylbenzene copolymers functionalized with aminobenzoic groups.

Polymeric Adsorbent	Surface Area, m^2^/g	Total Pore Volume, cm^3^/g
PAB1	20.14	2.44 × 10^−2^, for pores with ϕ < 45.7 nm
PAB2	27.89	4.11 × 10^−2^, for pores with ϕ < 47.2 nm
PAB3	34.56	5.68 × 10^−2^, for pores with ϕ < 47.6 nm

**Table 3:** Separation factors *R_L_* for tetracycline adsorption onto PAB1, PAB2, and PAB3 adsorbents.

Temperature (K)	*C_o_* (mM·L^−1^)	*R_L_* (PAB1)	*R_L_* (PAB2)	*R_L_* (PAB3)
298	3.000	0.367	0.257	0.155
	2.500	0.411	0.293	0.180
	2.000	0.466	0.341	0.215
	1.000	0.635	0.509	0.354
308	3.000	0.391	0.285	0.195
	2.500	0.435	0.324	0.225
	2.000	0.491	0.375	0.266
	1.000	0.658	0.545	0.420
313	3.000	0.423	0.307	0.230
	2.500	0.468	0.348	0.264
	2.000	0.524	0.400	0.310
	1.000	0.687	0.571	0.473
318	3.000	0.438	0.337	0.262
	2.500	0.484	0.379	0.299
	2.000	0.539	0.433	0.347
	1.000	0.701	0.604	0.515

## Data Availability

Not applicable.

## Supplementary Materials

The following supporting information can be downloaded at: https://www.mdpi.com/article/10.3390/molecules27092894/s1. Figure S1: Chemical structures of the adsorbates. Table S1: Kinetic parameters of pseudo-first-order (PFO) and pseudo-second-order (PSO) for the adsorption of sulfamethoxazole and tetracycline on PAB 1, PAB 2, and PAB 3 polymeric adsorbents. Table S2: Experimental equilibrium adsorption data. Table S3: Parameters of Langmuir, Freundlich, Sips, and Redlich–Peterson adsorption isotherms in the adsorption of sulfamethoxazole. Table S4: Separation factors RL for sulfamethoxazole adsorption onto PAB1, PAB2, and PAB3 adsorbents. Table S5: Parameters of Langmuir, Freundlich, Sips, and Redlich–Peterson adsorption isotherms in the adsorption of tetracycline.

## Sample Availability

Samples of the compounds PAB1, PAB2, and PAB 3 are available from the authors.
