# Supplementary material for: New Polymeric Adsorbents Functionalized with Aminobenzoic Groups for the Removal of Residual Antibiotics
Source: Molecules. 2022 Apr 30;27(9):2894. doi: 10.3390/molecules27092894 (PMC9101298; doi:10.3390/molecules27092894)
Supplement: Supplementary file 1 [file molecules-27-02894-s001.zip › molecules-1686978-SI.pdf]

molecules

Supplementary Materials

New Polymeric Adsorbents Functionalized with Aminobenzoic Groups for Removal of Residual Antibiotics

Radu Ardelean[1], Adriana Popa[2], Ecaterina Stela Drăgan[3*], Corneliu-Mircea Davidescu[1,4*] and Maria Ignat[5]

Table S1. Kinetic parameters of pseudo-first order (PFO) and pseudo-second order (PSO) for the adsorption of sulfamethoxazole and tetracycline on PAB 1, PAB 2 and PAB 3 polymeric adsorbents.

Table S2. Experimental equilibrium adsorption data.

Table S3. Parameters of Langmuir, Freundlich, Sips and Redlich-Peterson adsorption isotherms in the adsorption of Sulfamethoxazole.

Table S4. Separation factors R_L for sulfamethoxazole adsorption onto PAB1, PAB2 and PAB3 adsorbents.

Table S5. Parameters of Langmuir, Freundlich, Sips and Redlich-Peterson adsorption isotherms in the adsorption of Tetracycline.

Figure S1. Chemical structures of the adsorbates.

Table S1. Kinetic parameters of pseudo-first order (PFO) and pseudo-second order (PSO) for the adsorption of sulfamethoxazole and tetracycline on PAB 1, PAB 2 and PAB 3 polymeric adsorbents.

Polymeric adsorbent	Adsorbate	Temperature, [K]	PFO		PSO	
			k_1, [h^{-1}]	R^2	k_2 [$L.h^{-1}.mM^{-1}$]	R^2
PAB 1	sulfamethoxazole	298	0.434	0.9572	3.803	0.9920
		308	0.484	0.9729	6.067	0.9961
		313	0.493	0.9614	8.792	0.9978
		318	0.478	0.9824	11.444	0.9990
	tetracycline	298	0.529	0.9875	4.440	0.9968
		308	0.545	0.9876	6.002	0.9984
		313	0.551	0.9874	8.052	0.9991
		318	0.593	0.9774	10.992	0.9996
PAB 2	sulfamethoxazole	298	0.436	0.9842	4.935	0.9971
		308	0.519	0.9809	6.384	0.9977
		313	0.516	0.9841	9.202	0.9989
		318	0.471	0.9697	11.216	0.9994
	tetracycline	298	0.524	0.9854	3.716	0.9963
		308	0.549	0.9802	4.968	0.9979
		313	0.525	0.9865	6.523	0.9989
		318	0.560	0.9788	8.078	0.9991
PAB 3	sulfamethoxazole	298	0.481	0.9709	5.459	0.9979
		308	0.520	0.9762	6.328	0.9986
		313	0.544	0.9851	9.368	0.9991
		318	0.572	0.9701	11.064	0.9994
	tetracycline	298	0.543	0.9866	3.701	0.9975
		308	0.557	0.9801	4.808	0.9984
		313	0.586	0.9815	5.853	0.9988
		318	0.630	0.9862	7.713	0.9991

Table S2. Experimental equilibrium adsorption data.

Adsorbent	Adsorbate	Temperature							
	Sulfa-methoxazole	298 [K]		308 [K]		313 [K]		318 [K]	
	C_i [mM/L]	C_e [mM/L]	q_e [mM/g]	C_e [mM/L]	q_e [mM/g]	C_e [mM/L]	q_e [mM/g]	C_e [mM/L]	q_e [mM/g]
PAB1	3.000	1.988	0.127	2.017	0.123	2.038	0.120	2.058	0.118
	2.500	1.633	0.108	1.659	0.105	1.679	0.103	1.697	0.100
	2.000	1.287	0.089	1.309	0.086	1.326	0.084	1.342	0.082
	1.000	0.622	0.047	0.635	0.046	0.645	0.044	0.655	0.043
	Tetracycline	298 [K]		308 [K]		313 [K]		318 [K]	
	3.000	1.301	0.212	1.327	0.209	1.344	0.207	1.357	0.205
	2.500	1.033	0.183	1.057	0.180	1.075	0.178	1.089	0.176
	2.000	0.782	0.152	0.805	0.149	0.821	0.147	0.835	0.146
	1.000	0.341	0.082	0.356	0.081	0.368	0.079	0.377	0.078
PAB2	Sulfa-methoxazole	298 [K]		308 [K]		313 [K]		318 [K]	
	C_i [mM/L]	C_e [mM/L]	q_e [mM/g]	C_e [mM/L]	q_e [mM/g]	C_e [mM/L]	q_e [mM/g]	C_e [mM/L]	q_e [mM/g]
	3.000	1.704	0.162	1.727	0.159	1.746	0.157	1.766	0.154
	2.500	1.388	0.139	1.409	0.136	1.427	0.134	1.446	0.132
	2.000	1.083	0.115	1.102	0.112	1.118	0.110	1.135	0.108
	1.000	0.511	0.061	0.523	0.060	0.533	0.058	0.544	0.057
	Tetracycline	298 [K]		308 [K]		313 [K]		318 [K]	
	3.000	1.128	0.234	1.151	0.231	1.170	0.229	1.197	0.225
	2.500	0.877	0.203	0.900	0.200	0.919	0.198	0.946	0.194
	2.000	0.647	0.169	0.669	0.166	0.688	0.164	0.713	0.161
	1.000	0.261	0.092	0.277	0.090	0.290	0.089	0.308	0.087
PAB3	Sulfa-methoxazole	298 [K]		308 [K]		313 [K]		318 [K]	
	C_i [mM/L]	C_e [mM/L]	q_e [mM/g]	C_e [mM/L]	q_e [mM/g]	C_e [mM/L]	q_e [mM/g]	C_e [mM/L]	q_e [mM/g]
	3.000	1.457	0.193	1.480	0.190	1.492	0.189	1.519	0.185
	2.500	1.175	0.166	1.197	0.163	1.210	0.161	1.234	0.158
	2.000	0.907	0.137	0.927	0.134	0.939	0.133	0.961	0.130
	1.000	0.417	0.073	0.430	0.071	0.438	0.070	0.452	0.069
	Tetracycline	298 [K]		308 [K]		313 [K]		318 [K]	
	3.000	0.697	0.288	0.728	0.284	0.748	0.282	0.761	0.280
	2.500	0.528	0.247	0.559	0.243	0.579	0.240	0.596	0.238
	2.000	0.377	0.203	0.406	0.199	0.427	0.197	0.444	0.195
	1.000	0.140	0.108	0.16	0.105	0.175	0.103	0.187	0.102

Table S3. Parameters of Langmuir, Freundlich, Sips and Redlich-Peterson adsorption isotherms in the adsorption of Sulfamethoxazole.

Adsorption isotherm	Parameter	Temperature [K]	Adsorbent		
			PAB1	PAB2	PAB3
Langmuir	K_L [L/mM]	298	0.143±0.006	0.239±0.004	0.347±0.008
		308	0.141±0.007	0.220±0.010	0.302±0.007
		313	0.123±0.005	0.187±0.004	0.274±0.011
		318	0.109±0.005	0.178±0.003	0.259±0.009
	q_{max} [mM/g]	298	0.571±0.019	0.556±0.007	0.573±0.010
		308	0.553±0.024	0.575±0.019	0.614±0.010
		313	0.598±0.019	0.638±0.012	0.648±0.020
		318	0.645±0.025	0.643±0.010	0.654±0.017
	R^2	298	0.99996	0.99999	0.99997
		308	0.99994	0.99994	0.99998
		313	0.99997	0.99999	0.99994
		318	0.99997	0.99999	0.99996
Freundlich	K_F [$mM^{(1-1/n)}L^{1/n}g^{-1}$]	298	0.071±0.001	0.106±0.001	0.146±0.001
		308	0.068±0.001	0.103±0.001	0.141±0.001
		313	0.065±0.001	0.099±0.001	0.138±0.001
		318	0.063±0.001	0.096±0.001	0.133±0.001
	$1/n$	298	0.847±0.012	0.799±0.018	0.766±0.018
		308	0.846±0.008	0.808±0.012	0.785±0.017
		313	0.863±0.016	0.830±0.014	0.799±0.016
		318	0.875±0.009	0.835±0.015	0.804±0.014
	R^2	298	0.99984	0.99958	0.99951
		308	0.99991	0.99981	0.99959
		313	0.99968	0.99977	0.99965
		318	0.99990	0.99971	0.99973
Sips	K_S [L/mM]	298	0.106±0.024	0.216±0.018	0.280±0.001
		308	0.081±0.009	0.142±0.003	0.247±0.007
		313	0.143±0.025	0.151±0.005	0.212±0.047
		318	0.077±0.019	0.165±0.019	0.189±0.009
	q_m [mM/g]	298	0.745±0.149	0.606±0.041	0.674±0.003
		308	0.914±0.094	0.834±0.017	0.718±0.016
		313	0.526±0.079	0.761±0.021	0.797±0.014
		318	0.888±0.208	0.687±0.066	0.842±0.038
	n	298	0.969±0.026	0.980±0.015	0.955±0.001
		308	0.932±0.009	0.930±0.003	0.961±0.005
		313	1.022±0.029	0.967±0.004	0.954±0.033
		318	0.961±0.022	0.987±0.017	0.947±0.007
	R^2	298	0.99998	0.99999	1.00000
		308	1.00000	1.00000	1.00000
		313	0.99997	1.00000	0.99996
		318	0.99998	0.99999	1.00000

Table S3. *Cont.*

Adsorption isotherm	Parameter	Temperature [K]	Adsorbent		
			PAB1	PAB2	PAB3
Redlich-Peterson	K_{RP} [L/mM]	298	0.087±0.004	0.137±0.002	0.211±0.001
		308	0.088±0.003	0.141±0.001	0.195±0.002
		313	0.072±0.002	0.124±0.001	0.189±0.010
		318	0.074±0.003	0.116±0.003	0.182±0.002
	a $[(L/mM)^b]$	298	0.215±0.056	0.271±0.025	0.429±0.006
		308	0.288±0.043	0.365±0.003	0.369±0.013
		313	0.096±0.024	0.241±0.007	0.361±0.075
		318	0.175±0.053	0.194±0.027	0.359±0.015
	b	298	0.760±0.134	0.913±0.058	0.850±0.008
		308	0.620±0.062	0.700±0.004	0.855±0.023
		313	0.995±0.186	0.832±0.017	0.807±0.125
		318	0.721±0.151	0.939±0.093	0.777±0.023
	R^2	298	0.99998	0.99999	1.00000
		308	1.00000	1.00000	1.00000
		313	0.99997	1.00000	0.99998
		318	0.99998	0.99999	1.00000

Table S4. Separation factors R_L for sulfamethoxazole adsorption onto PAB1, PAB2 and PAB3 adsorbents.

Temperature [K]	C_o [mM.L^{-1}]	R_L PAB1	R_L PAB2	R_L PAB3
298	3.000	0.700	0.582	0.490
	2.500	0.737	0.626	0.535
	2.000	0.778	0.677	0.590
	1.000	0.875	0.807	0.742
308	3.000	0.703	0.602	0.525
	2.500	0.739	0.645	0.570
	2.000	0.780	0.694	0.623
	1.000	0.876	0.820	0.768
313	3.000	0.730	0.641	0.549
	2.500	0.765	0.681	0.593
	2.000	0.803	0.728	0.646
	1.000	0.890	0.842	0.785
318	3.000	0.754	0.652	0.563
	2.500	0.786	0.692	0.607
	2.000	0.821	0.737	0.659
	1.000	0.902	0.849	0.794

Table S5. Parameters of Langmuir, Freundlich, Sips and Redlich-Peterson adsorption isotherms in the adsorption of Tetracycline.

Adsorption isotherm	Parameter	Temperature, [K]	Adsorbent		
			PAB1	PAB2	PAB3
Langmuir	K_L [L/mM]	298	0.574±0.028	0.965±0.072	1.821±0.199
		308	0.519±0.032	0.835±0.056	1.380±0.118
		313	0.455±0.021	0.751±0.050	1.115±0.078
		318	0.427±0.017	0.656±0.036	0.940±0.066
	q_{max} [mM/g]	298	0.493±0.015	0.445±0.018	0.509±0.029
		308	0.510±0.021	0.468±0.018	0.562±0.028
		313	0.543±0.017	0.486±0.019	0.616±0.026
		318	0.556±0.016	0.509±0.018	0.687±0.030
	R^2	298	0.99983	0.99983	0.99961
		308	0.99975	0.99959	0.99929
		313	0.99987	0.99963	0.99961
		318	0.99991	0.99977	0.99966
Freundlich	K_F [$mM^{(1-1/n)}L^{1/n}g^{-1}$]	298	0.178±0.001	0.219±0.002	0.361±0.004
		308	0.172±0.001	0.213±0.002	0.351±0.004
		313	0.168±0.001	0.208±0.002	0.346±0.004
		318	0.164±0.001	0.200±0.001	0.342±0.003
	$1/n$	298	0.698±0.018	0.628±0.017	0.602±0.014
		308	0.712±0.015	0.651±0.017	0.646±0.016
		313	0.734±0.016	0.668±0.017	0.681±0.017
		318	0.743±0.018	0.689±0.018	0.708±0.015
	R^2	298	0.99942	0.99935	0.99952
		308	0.99963	0.99937	0.99945
		313	0.99957	0.99946	0.99941
		318	0.99952	0.99944	0.99957
Sips	K_S [L/mM]	298	0.359±0.016	0.449±0.004	0.513±0.005
		308	0.275±0.007	0.428±0.007	0.539±0.004
		313	0.289±0.003	0.383±0.020	0.541±0.052
		318	0.299±0.035	0.381±0.015	0.543±0.053
	q_m [mM/g]	298	0.678±0.021	0.706±0.001	1.023±0.008
		308	0.803±0.015	0.710±0.008	0.965±0.005
		313	0.754±0.006	0.752±0.028	0.949±0.062
		318	0.720±0.064	0.729±0.021	1.07±0.093
	n	298	0.892±0.008	0.817±0.001	0.747±0.001
		308	0.869±0.004	0.843±0.003	0.811±0.001
		313	0.906±0.002	0.848±0.009	0.856±0.015
		318	0.925±0.021	0.877±0.007	0.862±0.016
	R^2	298	1.00000	1.00000	1.00000
		308	1.00000	1.00000	1.00000
		313	1.00000	1.00000	0.99999
		318	0.99998	1.00000	0.99999

Table S5. *Cont.*

Adsorption isotherm	Parameter	Temperature, [K]	Adsorbent		
			PAB1	PAB2	PAB3
Redlich-Peterson	K_{RP} [L/mM]	298	0.332±0.003	0.588±0.008	1.590±0.027
		308	0.331±0.006	0.506±0.009	1.106±0.014
		313	0.285±0.001	0.471±0.016	0.878±0.024
		318	0.264±0.009	0.403±0.003	0.801±0.029
	a [$(L/mM)^b$]	298	0.856±0.019	1.689±0.038	3.558±0.074
		308	0.909±0.037	1.378±0.044	2.256±0.038
		313	0.684±0.004	1.262±0.078	1.622±0.059
		318	0.594±0.055	1.007±0.016	1.410±0.084
	b	298	0.747±0.011	0.690±0.008	0.614±0.006
		308	0.672±0.016	0.704±0.013	0.648±0.007
		313	0.738±0.003	0.691±0.025	0.685±0.022
		318	0.777±0.052	0.725±0.075	0.658±0.030
	R^2	298	1.00000	1.00000	1.00000
		308	1.00000	1.00000	1.00000
		313	1.00000	0.99999	0.99999
		318	0.99999	0.99999	0.99999

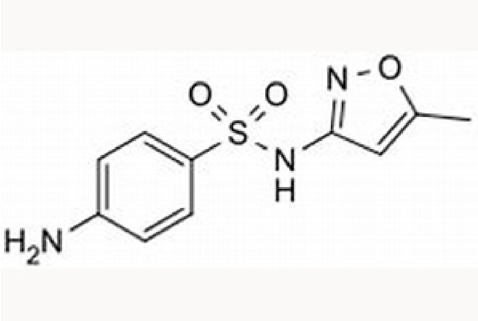

Sulfamethoxazole

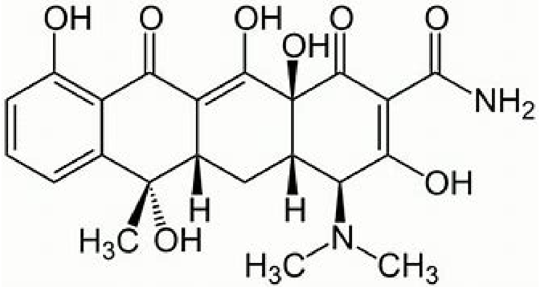

Tetracycline

Figure S1. Chemical structures of the adsorbates.
